# Opportunistic salpingectomy for ovarian cancer prevention

**DOI:** 10.1186/s40661-015-0014-1

**Published:** 2015-09-17

**Authors:** Gillian E. Hanley, Jessica N. McAlpine, Janice S. Kwon, Gillian Mitchell

**Affiliations:** Department of Gynaecology and Obstetrics, Division of Gynaecologic Oncology, University of British Columbia, Vancouver, BC Canada; Hereditary Cancer Program, BC Cancer Agency, Vancouver, BC Canada; Department of Medicine, University of British Columbia, Vancouver, BC Canada

**Keywords:** Ovarian cancer, Bilateral salpingectomy, Cancer prevention, Fallopian tube

## Abstract

Recently accumulated evidence has strongly indicated that the fallopian tube is the site of origin for the majority of high-grade serous ovarian or peritoneal carcinomas. As a result, recommendations have been made to change surgical practice in women at general population risk for ovarian cancer and perform bilateral salpingectomy at the time of hysterectomy without oophorectomy and in lieu of tubal ligation, a practice that has been termed opportunistic salpingectomy (OS). Despite suggestions that bilateral salpingectomy may be used as an interim procedure in women with BRCA1/2 mutations, enabling them to delay oophorectomy, there is insufficient evidence to support this practice as a safe alternative and risk-reducing bilateral salpingo-oophorectomy remains the recommended standard of care for high-risk women. While evidence on uptake of OS is sparse, it points toward increasing practice of OS during hysterectomy. The practice of OS for sterilization purposes, although expanding, appears to be less common. Operative and perioperative complications as measured by administered blood transfusions, hospital length of stay and readmissions were not increased with the addition of OS either at time of hysterectomy or for sterilization. Additional operating room time was 16 and 10 min for OS with hysterectomy and OS for sterilization, respectively. Short-term studies of the consequences of OS on ovarian function indicate no difference between women undergoing hysterectomy alone and hysterectomy with OS, but no long-term data exist. There is emerging evidence of effectiveness of excisional sterilization on reducing ovarian cancer rates from Rochester (OR = 0.36 95 % CI 0.13, 1.02), and bilateral salpingectomy from Denmark (OR = 0.58 95 % CI 0.36, 0.95) and Sweden (HR = 0.35, 95 % CI 0.17, 0.73), but these studies suffer from limitations, including that they were performed for pathological rather than prophylactic purposes. Initial cost-effectiveness modeling indicates that OS is cost-effective over a wide range of costs and risk estimates. While preliminary safety, efficacy, and cost-effectiveness data are promising, further research is needed (particularly long-term data on ovarian function) to firmly establish the safety of the procedure. The marginal benefit of OS compared with tubal ligation or hysterectomy alone needs to be established through large prospective studies of OS done for prophylaxis.

## Introduction

Ovarian cancer is the leading cause of death due to gynecologic malignancy and the fifth most common cause of cancer deaths in developed countries. In the United States (US) and Canada, there are ~25,000 new diagnoses and ~16,000 deaths from the disease annually. While the general population lifetime risk of ovarian cancer is 1.4 % [1], women at high-risk of developing the disease due to their inheritance of a germline BRCA1 and BRCA2 mutation have an average cumulative risk of between 40 % to 75 % and 8 % to 34 %, respectively [2–5]. Inherited germline mutations of BRCA1 and BRCA2 account for approximately 11.7 to 15 % of all invasive ovarian carcinomas [6–9]. In both general population and high-risk women, screening for ovarian cancer is not recommended, as no mortality benefit has been demonstrated even with strict adherence to screening protocols [10–14]. Symptoms of ovarian cancer are non-specific and often do not arise until the cancer is in a late stage, the point at which the majority of women are diagnosed [15]. Five-year overall survival is less than 50 % and has not substantially changed in the last two decades [16, 17].

Ovarian cancer is a heterogeneous disease and itscellular origins remain an area of active debate [18, 19]. It has been postulated that ovarian cancers can arise from the ovarian surface epithelium, fallopian tube epithelium and ectopic endometrium and it appears likely that different histological subtypes have different origins. There are five main histological subtypes of ovarian carcinoma: high-grade serous (HGSC), low-grade serous (LGSC), endometrioid (ENOC), clear cell (CCOC) and mucinous cancers. Each has a distinct clinical characteristics and genetic landscape [20]. HGSC is the most common, accounting for approximately 70 % of invasive ovarian carcinomas [21]. It is usually diagnosed at an advanced stage, and, although it is initially highly responsive to chemotherapy, most women with HGSC ultimately relapse, develop resistance to chemotherapeutic agents and succumb to their disease.

## Review

### A new understanding of the role of the fallopian tube in ovarian cancer

The ovary is the most frequent site of the dominant tumor mass at the time of cancer diagnosis, and this, together with the epidemiological evidence that increasing parity is strongly related to a reduction in ovarian carcinoma risk led to the “incessant” ovulation hypothesis for the etiology of ovarian cancer [22] and to the focus on Mullerian-type cortical inclusion cysts (Mullerian-CICs) within the ovary as the probable source of the disease. Mullerian-CICs were postulated to arise from transformation of the ovarian surface epithelium (OSE) trapped within the ovary after ovulation. The first suggestion of fallopian tube involvement in ovarian cancer was made as early as 1896, with the case report of a primary fallopian tube cancer with pathological characteristics very similar to ovarian cancer [23]. More recently, examination of the fallopian tubes removed at risk-reducing bilateral salpingo-oophorectomy (RRBSO) from women with BRCA1 and BRCA2 mutations revealed the presence in the distal fallopian tube (the fimbriae) of occult/small cancers in 5–15 % of these high-risk women [24–26] and preinvasive lesions in the fimbriae (serous tubal intraepithelial cancers; STICs) in 1–6 % of the women [27–30]. In contrast, only one paper that conducted intensive study of the ovaries found a single case (1 of 28 women studied (3.5 %)) [27] of premalignant epithelial change [25, 27, 31–37].

The Sectioning and Extensive Examining of the Fimbria (SEE-FIM) protocol was developed to maximize the detection of ovarian cancer precursors or early fallopian tube cancers by sectioning and examining the fallopian tube fimbriae for pathology [35, 38]. This protocol has revealed tubal involvement in up to 70 % of unselected women diagnosed with ovarian or primary peritoneal HGSC (with and without BRCA 1/2) [18, 39–43], including the presence of fimbrial STICs in 40–60 % of these women [18, 43, 44]— a proportion that increased with more complete examination of the fallopian tube [36, 42]. Importantly, STICs were not observed in women with non-gynecologic or benign conditions [37]. Based upon these findings, it has been proposed that tubal neoplasia is the primary lesion in HGSC and that these lesions spread to the ovary and peritoneum [18, 40].

Lending support to the theory that STICs are the precursor lesion to HGSC is the finding of identical TP53 mutations in STICs and concomitant ovarian and/or peritoneal cancers [39, 45]. It has been suggested that even earlier fallopian tube lesions precede STICs in the fallopian tube. The most well studies of these precursors is the ‘p53 signature’, defined as a focus of 12 or more cells with normal morphology, primarily localized at the fimbriated end of the fallopian tube, but with strong p53 immunostaining. Over 90 % of STICs have p53 signatures; p53 signatures have been reported in direct association or contiguous with STICs, and p53 signatures share identical TP53 mutations with both STICs and invasive cancers, all of which strongly suggests a clonal relationship among these tissues [45–47]. These data, along with the findings from the SEE-FIM protocol, underscore the fallopian tube as a clear target for prevention.

## Current recommendations

In BRCA1/2 mutation carriers, RRBSO has been shown to be highly protective for ovarian cancer with a cancer risk reduction of 80 % and overall mortality reduction of 60 % following surgery, and is strongly recommended for prevention of ovarian cancers in this population [34, 48, 49]. Most high-risk women have been reported to experience a high quality of physical and mental well being following RRBSO, with significantly reduced cancer-related worries [50]. However, RRBSO is not recommended for the general population, as removal of the ovaries has been reported to be associated with increased total mortality, coronary heart disease, stroke, osteoporosis and colorectal cancer [51, 52]. While RRBSO has demonstrated a reduction in overall mortality in the high-risk population, prospective follow-up has been short (e.g. 6 years in Domchek et al.) [49] and longer follow-up will be necessary to ensure that non-cancer events do not ultimately overtake the overall mortality benefit from the cancer prevention effects. Bilateral salpingectomy may offer significant protection against ovarian cancer in the general population, and possibly in the high-risk population, while avoiding these downstream health risks.

Given the new understanding regarding the role of the fallopian tube in ovarian cancer, and the health risks associated with RRBSO which make it an inappropriate candidate for prevention in the general population, recommendations were made regarding the treatment of the fallopian tube in common gynecologic surgeries. In September 2010 the Ovarian Cancer Research team (OVCARE) recommended to all gynecologic surgeons in the province of British Columbia (BC) Canada that, when operating on women at general population risk for ovarian cancer, they should consider: 1) performing bilateral salpingectomy at the time of hysterectomy (even when the ovaries are being preserved); and 2) performing bilateral salpingectomy in place of tubal ligation for sterilization—herein referred to as opportunistic salpingectomy (OS). The surgical practice changes were presented as a cancer prevention strategy of unproven efficacy. The Society of Gynecologic Oncology of Canada acknowledged BC’s campaign and officially endorsed BC’s cancer prevention strategy in 2011 issuing a statement recommending that the “physician discuss the risks and benefits of bilateral salpingectomy with patients undergoing hysterectomy or requesting permanent irreversible contraception” [53]. Two years later the US Society for Gynecologic Oncology followed suit and made a similar recommendation [54]. Most recently the American College of Obstetricians and Gynecologists published a statement supporting the recommendation that “surgeons and patients discuss the potential benefits of the removal of fallopian tubes during hysterectomy in women at population risk of ovarian cancer who are not having an oophorectomy” and that “when counselling women about laparoscopic sterilization methods, clinicians can communicate that bilateral salpingectomy can be considered a method that provides effective contraception”. These recommendations were made because “prophylactic salpingectomy may offer clinicians the opportunity to prevent ovarian cancer in their patients” [55].

## Numbers of hysterectomies and tubal sterilizations in North America

There are approximately 430,000 and 41,000 hysterectomies performed annually in the US and Canada, respectively [56, 57]. In the US, between 50 and 55 % of women who had a hysterectomy also had a bilateral oophorectomy, while this number appears to be around 45 % of hysterectomies in Canada [58, 59]. This means that there are approximately 240,000 women of general ovarian cancer risk undergoing hysterectomies annually in the US and Canada who would likely be eligible for OS for ovarian cancer prevention.

Approximately 350,000 and 25,000 tubal sterilizations are done annually in the US and Canada, respectively [60, 61]. Approximately half of these annual tubal sterilizations occur after delivery, typically at the time of caesarean delivery or within 24 h after a vaginal delivery [62]. Salpingectomy as a primary method of sterilization has not been considered routinely until the past few years. However, for individuals in whom tubal sterilization fails, bilateral salpingectomy has long been considered the preferred method to ensure definitive treatment [63]. Combining these women with the women undergoing hysterectomy without oophorectomy results in approximately 590,000 women who are potentially eligible for OS for ovarian cancer prevention purposes annually in the US and Canada.

Women at high-risk for ovarian cancer (those with BRCA1 and BRCA1 mutations) are strongly advised to have prophylactic RRBSO once child-bearing is complete based on the good short term data indicating the improvement in mortality in this cohort [49]. However, the long-term effects of premature menopause on mortality and morbidity in this cohort are largely unknown, and concerns regarding these effects have led to discussion of a staged approach of initial bilateral salpingectomy once childbearing is complete, followed by an oophorectomy closer to natural menopause [64–66]. While this presents a potentially promising alternative to premature menopause and the resulting health consequences, we do not yet have the prospective evidence demonstrating that a staged approach is not inferior to upfront RRBSO. RRBSO has an important impact on breast cancer risk in this population; the 50 % reduction in breast cancer incidence associated with premenopausal RRBSO in high-risk women [49] would also need to be considered in these prospective studies before changing clinical practice for ovarian cancer prevention among BRCA1/2 mutation carriers.

## Opportunistic salpingectomy in the general population

### Uptake

The uptake of OS has been studied in depth in British Columbia where the campaign was first initiated and was then adopted across Canada more widely. To examine rates of OS in BC we examined all hospitalizations in the province using the Discharge Abstract Database, which captures demographic, administrative and clinical information for all hospital discharges (inpatient and days surgeries) [67] beginning from the calendar year two years prior the educational campaign (Sept 2010) and continuing two years after. We reported that the proportion of hysterectomies with an associated OS (excluding hysterectomies where ovaries were removed) increased from 8 % in 2008 to 63 % in 2011, and the proportion of sterilizations by salpingectomy increased from 0.5 % in 2008 to 33 % in 2011 [59]. We have recently extended this analysis to 2013 and found that 75 % of all hysterectomies without oophorectomy included a bilateral salpingectomy and 48 % of all sterilizations were done by salpingectomy in 2013. While the rate of uptake in the rest of Canada has not been as dramatic, rates of hysterectomy with OS are significantly increasing from less than 1 % of all hysterectomies in 2006 to more than 11 % in 2011 [68]. There is less known about uptake of OS in the United States and while a large nationally representative study is needed, there are indications that OS is being performed in parts of the United States [69]. We expect that rates of OS will increase in the US following the ACOG’s January 2015 recommendation to discuss opportunistic salpingectomy with patients undergoing hysterectomy or tubal sterilization [55].

There have also been several surveys assessing physician attitudes towards OS. A Canadian survey of obstetrician-gynecologists revealed that 90 % had heard of OS, but 37 % were unaware of the evidence supporting the hypothesis that HGSC originates in the fallopian tube and 38 % were unsure whether there would be any population benefit to performing OS [70]. A survey of physicians in American institutions with Obstetrics & Gynecology residency programs reported that 54 % of physicians perform OS with hysterectomy. The 46 % of physicians who did not commonly perform OS reported that they did not believe there was any benefit [71]. While 58 % of practitioners believed it was the most effective method of sterilization after age 35 they only chose this method in patients in whom a previous tubal sterilization has failed or because of tubal disease [71]. Finally, a similar survey of Irish Obstetricians and Gynecologists reported that 90 % would consider OS at the time of abdominal hysterectomy and 73 % would consider OS for female sterilization [72].

### Safety

The operative and perioperative complications of OS have been studied in British Columbia. We reported that OS with hysterectomy requires an additional 16 min of OR time while OS for sterilization requires an additional 10 min of OR time compared with hysterectomy alone and tubal ligation, respectively. We found no increased risks associated with OS when examining length of stay in hospital, or the likelihood of hospital readmission or blood transfusion—both of which were raised as concerns by gynecologic surgeons at the time of the educational campaign in BC [70]. The BC study indicated that OS was performed by open, laparoscopic, and vaginal routes, the latter of which accounted for 18 % of hysterectomies with OS. Compared with open approach, the vaginal approach for hysterectomy with OS was associated with significantly shorter length of stay in hospital and decreased risk for hospital readmission (OR = 0.51, 95%CI 0.37, 0.70) [59]. Vaginal approach for hysterectomy with OS appears to be both safe and feasible  making hysterectomy with OS an option in both high and low resource settings.

OS also eliminates the risk of subsequent hydrosalpinx and, in the case of tubal sterilization, ectopic pregnancy—an advantage over conventional tubal sterilization methods such as partial salpingectomy, banding or coagulation. Hydrosalpinx is the most frequent complication following hysterectomy without OS, and occurs in 35.5 % of patients requiring revision surgery in 7.8 % of patients [73, 74]. Other complications following retained tubes after hysterectomy and sterilization include pelvic inflammatory disease, salpingitis, benign fallopian tube tumors, and tube prolapse [75–80]—many of which are definitively treated with salpingectomy and could be avoided by performing OS at the time of hysterectomy and in lieu of tubal ligation. A concern raised regarding OS in lieu of tubal ligation is the inability to reverse the procedure for women who subsequently wish to regain their fertility who will then be reliant on an in vitro fertilization approach. Recommendations regarding tubal reversal surgery post TL versus proceeding to in vitro fertilization (IVF) vary greatly across the globe, often reflecting public health or insurance cost coverage (for surgical procedures, for IVF) and dependent on the presence of skilled surgeons who are willing to perform tubal microsurgery for reversals. The overall cost of a single IVF cycle compared with tubal microsurgery may be comparable but successful tubal reversal would allow multiple attempts at child bearing as compared to a single round of IVF. In areas where IVF coverage is free or heavily subsidized, or where women may have additional factors that would make natural conception challenging (eg. decreased ovarian reserve, male factor infertility) IVF may be the first choice of management thus her options would be no different than for women who had undergone OS.

Salpingectomy, when performed correctly, should not impact the ovarian blood supply and, therefore, should not have an impact on ovarian function (hormonal production, ovulation, age of menopause). Hysterectomy with ovarian conservation has been associated with decreased ovarian function [81] and earlier onset of menopause in prospective studies [81, 82]. Thus, studies examining ovarian function after hysterectomy with and without OS tend to examine differences between the groups according to OS status rather than differences from baseline. Encouragingly, a retrospective series involving ~160 premenopausal women who had total laparoscopic hysterectomy with or without bilateral salpingectomy showed small differences in ovarian sonographic and hormonal parameters from baseline in both groups and no difference between the groups. Anti-Mullerian hormone (AMH) levels (a measure of ovarian reserve) were slightly lower in both groups (which is consistent with the research reporting decreased ovarian function following hysterectomy) but the addition of salpingectomy to the procedure did not worsen the effect [83]. The lack of a hormonal difference between the groups was also reported in a recent pilot randomized controlled trial examining the short-term effects of salpingectomy during laparoscopic hysterectomy on ovarian reserve among thirty premenopausal women. Again AMH levels following surgery were lower from baseline in both groups, but there were no difference in postoperative AMH levels between the group randomized to undergo opportunistic salpingectomy at the time of hysterectomy and the group retaining their fallopian tubes [84]. The long-term effects, such as the timing of menopause, have not been analysed systematically after hysterectomy with OS or OS for sterilization. This requires further study, as it is possible that if OS reduces the age of menopause, the ovarian cancer mortality benefit may be entirely offset by the increase in all cause mortality from the earlier age of onset of menopause. While the short-term data indicating hormonal equivalence between the OS and hysterectomy alone is somewhat reassuring , no long-term studies have been published to date.

### Effectiveness

We have long had epidemiologic evidence supporting the importance of the fallopian tube in ovarian cancer in the form of the reduced risk associated with tubal ligation (TL). TL appears to decrease the risk of ovarian cancer by 29 % overall [85, 86], but there are differences in this effect across histologic subtype with the greatest reduction in risk found for ENOC (52 %), followed by CCOC (48 %), and a 20 % reduction in risk of HGSC [86]. There is also encouraging data on a small number of excisional tubal ligation cases (defined as complete salpingectomy, distal fimbriectomy, or partial salpingectomy) from Minnesota. Researchers from the Rochester epidemiology project reported a 64 % reduction in the risk of ovarian cancer after excisional tubal sterilization compared to those without sterilization or with non-excisional tubal sterilization (OR = 0.36, 95 % CI 0.13, 1.02) [69]. While this study was small and did not distinguish bilateral salpingectomy from other forms of excisional tubal sterilization, the results are promising.

Danish researchers used a national database to study the relationship between bilateral salpingectomy and ovarian cancer in a retrospective cohort study [87]. They reported that bilateral salpingectomy reduced the risk for ovarian cancer by 42 % (OR = 0.58, 95 % CI 0.36, 0.95); however, their distribution of ovarian cancers by histologic subtype revealed a much smaller than usual proportion of HGSCs (46 % versus the standard 70 %). This suggests the possibility of some form of contamination of the cases, which would likely have decreased their estimate of risk reduction.

The most recent, largest and most rigorous study of the relationship between ovarian cancer and bilateral salpingectomy to date was a population-based retrospective Swedish study using health registers incorporating more than 5.5 million women and 30,000 ovarian cancer cases [88]. The authors identified the four gynecologic surgical procedures of interest (hysterectomy, hysterectomy with concomitant BSO, salpingectomy, sterilization). There were so few hysterectomies with concomitant salpingectomies (*n* = 2646) that these women were excluded from analyses. The authors examined the potential impact of one- vs. two-sided salpingectomy, but for codes ocurring after 1997, the consistency in reporting one- or two-sided procedures was poor, so the study was restricted to the calendar years 1973 to 1996. While they were able to control for parity and education level, they did not control for use of oral contraceptive pills (an important protective factor) [89]. They reported that hysterectomy with BSO resulted in an almost complete risk cessation (HR = 0.06, 95 % CI 0.03 to 0.12). One-sided salpingectomy was associated with a reduction of risk of 29 % (HR = 0.71, 95 % CI–0.56 to 0.91) while bilateral salpingectomy was associated with a 65 % reduction in risk (HR = 0.35, 95 % CI 0.17, 0.73). They also reported a reduction in risk associated with hysterectomy alone (HR = 0.79, 95 % CI 0.70 to 0.89) [88].

While this study illustrates that women who have had a bilateral salpingectomy more than halved their risk for ovarian cancer, there are important limitations that need to be addressed in future research. The cohort of women undergoing bilateral salpingectomy was small (n = 3051) since bilateral salpingectomy was a fairly uncommon procedure and historically has not been performed for prophylactic purposes. Salpingectomy was done for the indications of hydrosalpinx, infections (primarily pelvic inflammatory disease), ectopic pregnancy, and endometriosis—all conditions resulting in considerable inflammation. Both PID and endometriosis are risk factors for ovarian cancer [90, 91], suggesting that the group of women who underwent salpingectomy in this historical cohort may have already been at increased risk for ovarian cancer. It is also plausible that salpingectomies performed for prophylactic reasons may confer more protection than those done for other indications, as surgeons will be more careful to remove the entire distal end of the fallopian tube. For both of these reasons, OS may be more protective against ovarian cancer than the results on bilateral salpingectomy reported by Falconer et al. suggest. They also report the reduction of risk conferred by bilateral salpingectomy compared with women unexposed to any of the gynecologic surgeries of interest. As recommendations suggest performing OS with hysterectomy or in lieu of tubal ligation and hysterectomy alone and tubal ligation both reduce ovarian cancer risk [85], it will be important to understand the marginal benefit of performing OS in terms of the additional cancer cases prevented. It is also important to note that none of the studies summarized above reports any data on BRCA1 or BRCA2 mutation carriers and the results should only be generalized to women at population-level risk for ovarian cancer.

### Cost-effectiveness

Given the 590,000 women undergoing hysterectomy without oophorectomy and tubal sterilization annually in the US and Canada, implications of widespread performance of OS on health care system costs warrant further study and concerns have been raised [92]. While an accurate understanding of the effectiveness of OS in preventing ovarian cancer, as well as data on the long-term risks associated with this procedure is imperative to understand the implications of OS on our health care systems, in the absence of these data, we have used a decision analytic model to estimate the cost-effectiveness of OS as an ovarian cancer prevention strategy for the general population [61]. Using the assumptions that OS, BSO, hysterectomy, and tubal ligation each confer a 50 %, 90 %, 20 %, and 30 % reduction in risk for ovarian cancer, OS was found to be cost-effective. This result held over a wide range of costs and risk estimates. The model reported that hysterectomy with OS was less costly than hysterectomy alone or with bilateral salpingo-oophorectomy (BSO) but more effective with average comparative life expectancy gains of 1 week and 2 months (in the absence of routine hormone replacement after BSO), respectively. For sterilization, OS was more costly than tubal ligation but more effective with an average life expectancy gain of 1 week. While these average life expectancy gains appear insignificant, it represents a very large gain for women who would have died prematurely as a result of ovarian cancer averaged across many women in the population who receive no gain as they were never going to be diagnosed with ovarian cancer. The average life expectancy gain of 1 week is comparable to that of cervical cancer screening every 2 years compared to every 5 years [93]. The model suggested that the number of hysterectomies with OS needed to prevent one case of ovarian cancer was 273 and the corresponding NNT for salpingectomies for sterilization was 366—numbers that are in line with the number needed to vaccinate against human papilloma virus of 324 to prevent one case of cervical cancer [94]. As we learn more about OS, this model will be updated and improved, but these preliminary results suggest that OS may be cost saving in the long-term.

## Conclusions

Our understanding of the pathogenesis of ovarian cancer has improved drastically with the understanding that HGSC can originate in the fallopian tube and, as a result, our approach to ovarian cancer prevention has fundamentally changed for women in the general population and is being challenged for women at high risk of developing the disease. For women at population risk of ovarian cancer, opportunistic salpingectomy presents a promising approach to reducing incidence and mortality from ovarian cancer, and recommendations to integrate it into routine gynecologic practice are increasingly common. While preliminary safety and efficacy data are very reassuring, there remain some unanswered questions. Specifically, we need more data on the impact of OS on ovarian function, which is being examined both through planned randomized controlled trials and a cohort study in BC in order to determine if OS accelerates menopause. In addition, the interaction of OS with other risk-reducing measures including oral contraceptive use will require a greater number of patients to define. OS remains an exciting ovarian cancer prevention strategy in women at general population risk for ovarian cancer undergoing routine gynecologic surgeries and is increasingly being performed vaginally, laparoscopically and robotically. To be clear, we are not advocating surgical intervention solely for the purposes of salpingectomy nor change in surgical approach if the planned route for the required gynecologic surgery cannot achieve salpingectomy.

For women at high risk of ovarian cancer, such as women with germline BRCA 1/2 mutations, who are advised to consider RRBSO from age 35, the possibility of ameliorating some of the effects of premature menopause by either bilateral salpingectomy alone or a staged approach of early bilateral salpingectomy followed by bilateral oophorectomy closer to the age of natural menopause, is attractive. This two-staged approach appears to be most effective in terms of quality-adjusted life expectancy, and is cost-effective [65] providing the tubal hypothesis of serous ovarian cancer is correct. Although most BRCA-associated ovarian cancers likely arise in the fallopian tube, there are four important reasons why oophorectomy, either concurrent with bilateral salpingectomy or delayed, is still recommended in this population: (1) some of these cancers still appear to originate in the ovary, (2) oophorectomy prior to menopause is known to reduce breast cancer risk in this high-risk population by 50 % [95], (3) even when incorporating the increased morbidity associated with surgical menopause, there is still a significant reduction in all-cause mortality associated with RRBSO among high-risk women [49], and (4) while bilateral salpingectomy may reduce ovarian cancer risk, the degree of protective effect on ovarian cancer is unknown. For these reasons we do not consider that it is yet appropriate to routinely advise young high-risk women (BRCA1 or 2 mutation carriers) to have bilateral salpingectomy as a prevention strategy either as a sole or staged procedure with a delayed oophorectomy; bilateral salpingo-oophorectomy on completion of childbearing still has to be the standard of care. However, bilateral salpingectomy may be an option for a well counselled woman if they are not yet prepared to undergo oophorectomy ie., vs. no intervention at all. Before bilateral salpingectomy, with or without, later bilateral oophorectomy can be routinely offered to high-risk women, we need to know that it is effective and does not abrogate the cancer incidence and mortality benefits proven for RRBSO. This will likely require an international effort over many years in order to recruit a large enough sample of these highly selected patients, most likely in the form of a registry rather than a randomised trial to compare outcomes between bilateral salpingectomy and RRBSO [96].

In summary, opportunistic salpingectomy is a safe intervention in the short term, when done concurrently with hysterectomy or instead of tubal ligation. It has the potential to reduce the incidence and mortality from ovarian cancer, and it may have an important role as a temporizing measure in high-risk women with BRCA mutations who are unwilling to undergo standard risk reducing surgery (bilateral salpingo-oophorectomy) at an early age. It will still be essential to evaluate long-term safety and efficacy outcomes to support the ongoing use of this intervention in the general population as well as the high-risk setting.

## Abbreviations

HGSC: High Grade Serous ovarian cancer
LGSC: Low Grade Serous ovarian cancer
ENOC: Endometrioid ovarian cancer
CCOC: Clear cell ovarian cancer
BC: British Columbia
RRBSO: Risk Reducing bilateral salpingo-oophorectomy
OS: Opportunistic Salpingectomy
BSO: Bilateral salpingo-oophorectomy
TL: Tubal ligation
IVF: In vitro fertilization
ACOG: American College of Obstetrics & Gynecology
STICs: Serous tubal intraepithelial cancers
